# Exploring outer space biophysical phenomena via SpaceLID

**DOI:** 10.1038/s41598-023-44729-9

**Published:** 2023-10-13

**Authors:** Shanshan Wang, Tao Wang, Xian Zeng, Xinyi Chu, Dongzhi Zhuoma, Yufen Zhao, Yu Zong Chen

**Affiliations:** 1 Qian Xuesen Collaborative Research Center of Astrochemistry and Space Life Sciences, Institute of Drug Discovery Technology, Ningbo University, Ningbo, 315211 China; 2 Department of Biological Medicines and Shanghai Engineering Research Center of Immunotherapeutics, Fudan University School of Pharmacy, Shanghai, 201203 China; 3 Medical College, Tibet University, Lhasa, 850000 China; 4 Department of Chemical Biology, College of Chemistry and Chemical Engineering, and The Key Laboratory for Chemical Biology of Fujian Province, Xiamen University, Xiamen, 361005 China; 5 Key Laboratory of Bioorganic Phosphorus Chemistry and Chemical Biology (Ministry of Education), Department of Chemistry, Tsinghua University, Beijing, 102206 China

**Keywords:** Biophysics, Physiology

## Abstract

Extensive investigations in outer space have revealed not only how life adapts to the space environment, but also that interesting biophysical phenomena occur. These phenomena affect human health and other life forms (animals, plants, bacteria, and fungi), and to ensure the safety of future human space exploration need to be further investigated. This calls for joint research efforts between biologists and physicists, as these phenomena present cross-disciplinary barriers. Various national organizations provide useful forums for bridging this gap. Additional discussion avenues and database resources are helpful for facilitating the interdisciplinary investigations of these phenomena. In this paper, we present the newly established Space Life Investigation Database (SpaceLID, https://bidd.group/spacelid/) which provides information about biophysical phenomena occurring in space. Examples obtained using the database are given while discussing the underlying causes of these phenomena and their implications for the physiology and health of life in space.

## Introduction

In the past two decades, over 1500 experiments have been conducted on the International Space Station (ISS) for biological, human health, and biotechnology investigations^1^. More space-based biomedical experiments have been carried out on space shuttles and satellites^2^. These experiments have revealed not only biological insights into how life adapts to the space environment^2^, but also interesting biophysical phenomena occurring in humans in outer space. Further research on these space environment induced phenomena sheds light on how life is influenced by and responds to them. For space exploration, results have profound implications on the maintenance of the physiology and health of life, and for the potential usage of biotechnology for mitigation procedures.

The relevant research is interdisciplinary in nature, with publications scattered across a wide spectrum of specialized journals (e.g., NPJ Microgravity, J Am Med Assoc Networks) and interdisciplinary journals (e.g., Proc Natl Acad Sci USA). Researchers who encounter or are interested in these phenomena may face substantial cross-disciplinary barriers. Biologists need to be equipped with physics knowledge for the recognition and understanding of biophysical phenomena from their space-based research. On the other hand, physicists need to be informed of these potentially interesting phenomena from biologist colleagues, and more broadly from numerous publications in journals across other disciplines.

Various organizations provide valuable forums for bridging this cross-disciplinary gap. Examples include NASA's biological and physical sciences division, the National Academies committee on biological and physical sciences in space, and the American society of gravitational and space research. In addition, there exist other informative discussion avenues and dedicated web-resources that help bridge the cross-disciplinary knowledge gap and facilitate more extensive collaborations. For this purpose, we present the new online Space Life Investigation Database^3^ (SpaceLID) that was built to provide information and tools to investigate space induced biophysical phenomena based on health investigations of astronauts in space stations. In addition, the online GeneLab^4^ Data System that contains spaceflight-related omics (genomics, transcriptomics, proteomics, metabolomics) data, complementing the information available on SpaceLID is described. The paper provides examples of biophysical phenomena studies that are found on SpaceLID.

## Databases

**Figure 1:**
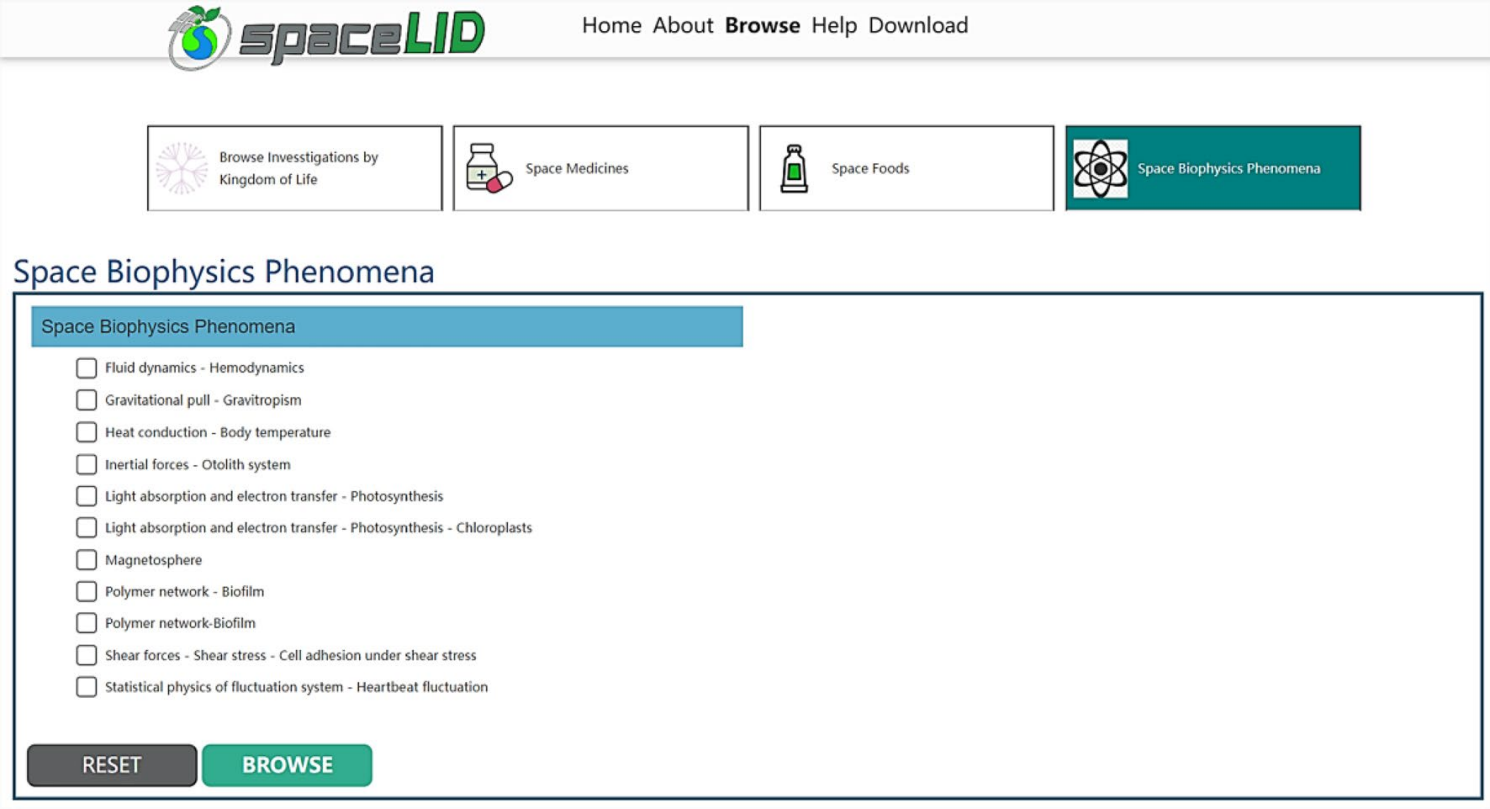
The Space Life Investigation Database (SpaceLID) and its Space Biophysics Phenomena search tool.

Analysis results of known biophysical phenomena occurring in astronauts in space may be easily retrieved from the newly-built Space Life Investigation Database (SpaceLID: https://bidd.group/spacelid/). The Wang et al. paper “Database of space life investigations and bioinformatics of microbiology in extreme environments”^3^ presents SpaceLID in general and describes its potential applications. SpaceLID is freely available online and covers three axes, respectively “Space Medicines”, “Space Foods”, and “Space Biophysics Phenomena”. The “Space Biophysics Phenomena” study search tool can be accessed via the Browse button on the SpaceLID homepage (Fig. 1). Each study includes a study description, investigation keywords with abstract, protocols followed, study outcomes, and associated publication(s). Biophysical phenomena covered by SpaceLID include fluid dynamics (hemodynamics), gravitational pull (gravitropism), heat conduction (body temperature), inertial forces (otolith system), light absorption and electron transfer (photosynthesis, chloroplasts), magnetosphere, polymer network (biofilm architecture, formation, and growth), shear forces (shear stress, cell adhesion under shear stress), and statistical physics of fluctuation system (heartbeat fluctuation). Overall, SpaceLID provides information on more than 450 space-based life investigations of 92 species (human, animal, plant, bacteria, and fungi), that were performed on various space missions.

Adding human health-related content in SpaceLID was a three-step process. Firstly, it was collected by searching PubMed articles using keyword combinations such as “spaceflight”, “International Space Station”, “ISS”, “space shuttle”, “satellite” and “human”, “astronaut”, “health”, “bone”, “blood”, “cardiovascular”, “liver”, “brain”, “skeletal”, “microbiota”, “urine”, “saliva”, “immune”, and “nervous”. Subsequently, these publications were manually checked to identify those containing experimental investigations of life in space. Those investigations that were found to include biophysical phenomena in space were then manually analyzed with particular focus on the investigations of the blood, cardiovascular system, gravitropism, body temperature, otolith system, photosynthesis, chloroplasts, magnetic effect, biofilm, and shear stress.

**Figure 2:**
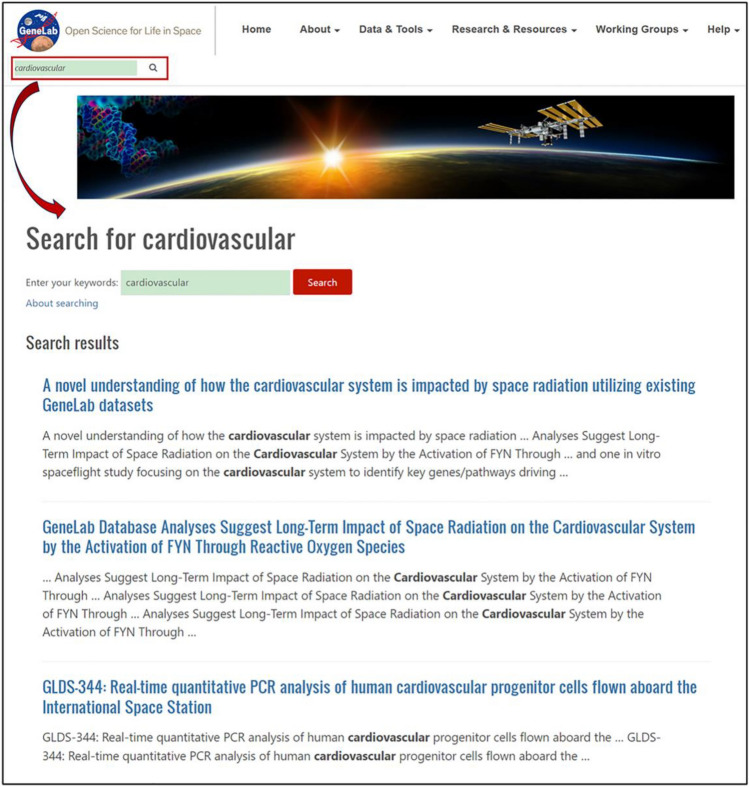
Relevant entries for cardiovascular search results in GeneLab.

The underlying mechanisms of biological responses to biophysics phenomena may be studied using the space-based omics data in the open access NASA Genelab database^4^ (https://genelab.nasa.gov/). Although the GeneLab is not yet established for cause-and-effect investigations, its enriched space-based omics data contain useful genetic, transcriptomic, and proteomic indications about how life is influenced and responds to the various biophysical phenomena in space. For instance, to investigate the cardiovascular response to heartbeat variations in space, one may input “cardiovascular” in the keyword search field of the Genelab homepage, which returns 3 relevant entries (Fig. 2). Two entries report the use of the Genelab omics data for the novel understanding of how the cardiovascular system is impacted by space radiation^5^. Based on the transcriptome profiling data of human umbilical vein endothelial cells and murine cardiomyocytes, the study found a gene FYN as the central driver/hub for the cardiovascular response to space radiation. The third entry provides a qPCR gene expression dataset of human cardiovascular progenitor cell clonal populations. These data may be further explored for molecular mechanisms of the response to the varied power-law correlation of the human heartbeat.

## Results and discussion

To show how SpaceLID can be used by the scientific community, we present below two examples illustrating biophysical phenomena that astronauts encounter during space missions. Retrieved via the “Space Biophysics Phenomena” study search tool, these examples cover the effects and accompanying analysis results on the core body temperature, heart rate, and intrinsic autonomic regulatory system. The “search terms” that were used to find the Study ID publications are given so that the reader can reproduce the search results. For each example, relevant references from the “Space Biophysics Phenomena” search are indicated in the text along with supporting literature.

**Figure 3:**
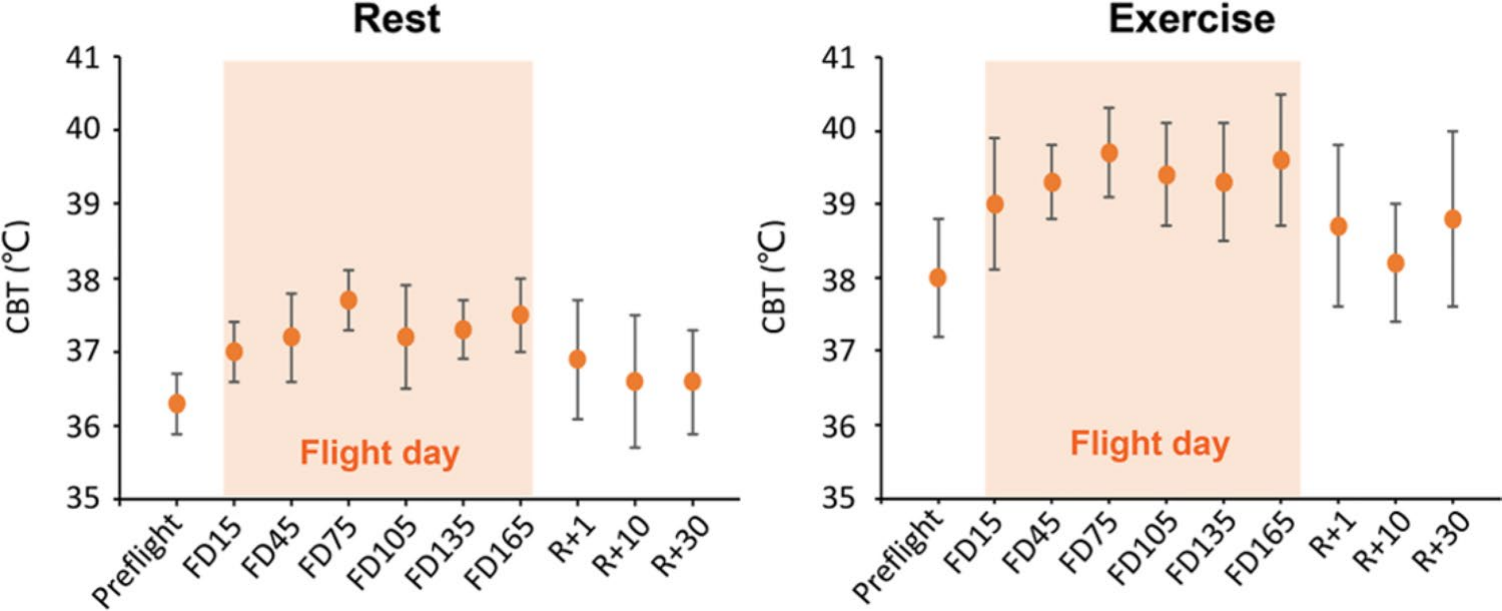
The mean core body temperature (CBT) of 11 astronauts at rest (left panel) and during exercise (right panel) on the ISS. Figure reproduced from Fig. 1 of Ref.^7^.

The first example concerns the increased core body temperature (CBT) of astronauts during long-duration spaceflight, which is a prominent biophysical phenomenon in space. CBT regulation relies on heat transfer via thermal radiation, convection, and evaporation. By using the “Space Biophysics Phenomena” study search tool (search term: “Heat conduction—Body temperature”), 4 studies were retrieved. The mean CBT of 2 astronauts on the Russian MIR space station, estimated by the morning foot temperature, is up to 3 °C higher than what it is on the ground^6^. The mean CBT of 11 astronauts at rest and during exercises on the ISS, measured by a heat flux sensor positioned at the forehead, is about 1.2 °C and 1.7 °C higher than the respective values on the ground (Fig. 3), and their CBT may increase to 39–40 °C during exercises on the ISS^7^. It is noted that thermography may not be the best diagnostic tool for monitoring CBT. Even so, these measurements may still reflect larger variations of CBT. The increased CBT is primarily due to reduced convective and evaporative heat-loss of the human body in space^7–9^. An experiment has shown that the average evaporation rate of hydrofluoroether (HFE-7100) sessile drops under microgravity are 45% and 56% lower than that on the ground with and without the electric field respectively^8^.

Under the influence of microgravity in space, the surface of the human body changes from a chemodynamic motor state to a thermal one, with a double decrease in Carnot’s efficiency^6^. The human body responds to this reduced heat-loss by peripheral vasodilation, which subsequently increases skin perfusion for reinforced radiative heat-loss^10^. This intricate heat-loss response is distorted during exercise in space when > 80% of the energy expenditure is converted to heat, leading to a marked increase in CBT. These observations are consistent with the anecdotal evidence from astronauts complaining about thermal discomfort^6,9^, and with the reporting that heat-related challenges are critical issues during physical exercise in weightlessness^11^.

**Figure 4:**
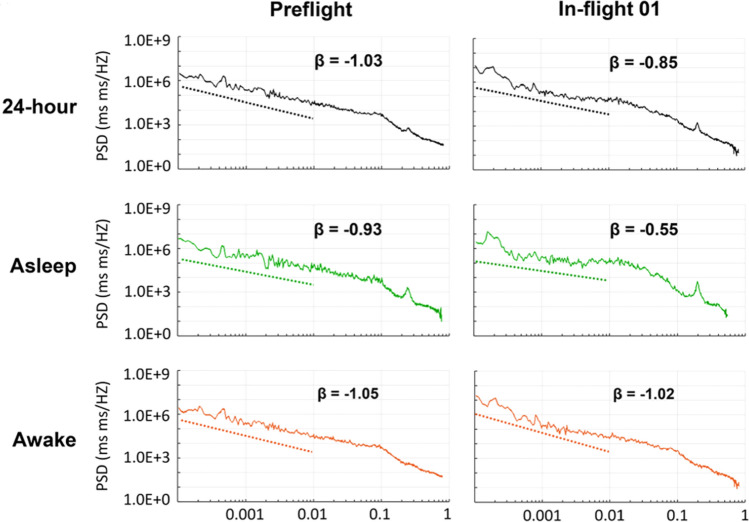
Power spectra of long-term heart rate variability of 7 astronauts on the ISS in the frequency range of 0.0001–0.01 Hz (periods of 2.8 h to 1.6 min). Left and right: cardiovascular fractal dynamics assessed by the slope of fractal scaling β before spaceflight and during spaceflight (days 24 ± 5 of flight). Top, middle, and bottom row: 24-h span, sleep span, and awake span. Figure reproduced from Fig. 3 of Ref.^12^.

In the second example, the effects of the space environment on the heart rate and intrinsic autonomic regulatory system are investigated. By using the “Space Biophysics Phenomena” study search tool (search term: “Statistical physics of fluctuation system—Heartbeat fluctuation”), 9 studies were retrieved. The interplay between human response activities and the variable fluid dynamics in space influences cardiovascular regulation, leading to the alteration of another biophysical phenomenon in space^12^. Healthy living systems are self-regulated to reduce variability and maintain homeostasis. Statistical physics studies have revealed the presence of long-range power-law correlations in a wide variety of systems, including human heartbeat fluctuations^13^. In biomedical research, heart rate variability has primarily been studied by various linear and non-linear methods^14^. It has been found that investigations from the power-law perspective expose important insights about heartbeat fluctuations^13^. The observed power-law correlation in space implies that the heartbeat at a given time is related not only to the immediately preceding heartbeat but to fluctuations in the remote past. The fractal dynamics of the power-law correlations can be assessed by the slope of the scaling β. The scaling β of the heartbeat of 7 astronauts is slightly reduced during a long-duration spaceflight on the ISS (Fig. 4)^12^, which indicates a reduced long-range correlation of heartbeat.

Investigations have shown that the spectral region most affected by microgravity is the ultra-low frequency (ULF) spectral power, and the decrease in ULF power is primarily due to changes in two ULF bands^12^. One is associated with behavior independent of the intrinsic autonomic regulatory system^15,16^. Another is partly related to the intrinsic autonomic regulatory system (0.001–0.003 Hz) and partly to the very low frequency (VLF) component (0.003–0.005 Hz). These results suggest that exposure to microgravity affects the intrinsic autonomic regulatory system of the astronauts throughout spaceflight. Certain pathological conditions such as Huntington’s disease are marked by diminished power-law correlations^12^. Further investigation and research are necessary to understand the broader health risks associated with the altered power-law correlation and to develop appropriate countermeasures to mitigate any potential adverse effects on astronauts’ well-being.

## Summary

In addition to the biophysical phenomena in space that have been presented, there are also other space environment-induced effects. For instance, astronauts under weightless conditions experience a sustained redistribution of fluids towards the head, while the hydrostatic effects of this redistribution lead to changed venous pressure and sustained blood flow stagnation with increased thrombus risk^17^. The fluid shift may also cause changes to the brain structure, such as an upward shift of the brain^18^, and alterations in the microstructure of brain white matter^19^, affecting communication and information transmission between neurons, subsequently influencing cognitive function. Furthermore, fluid shifts can potentially lead to increased intracranial pressure, impacting ocular structures and visual function^20^, which is associated with spaceflight-associated ophthalmic changes. Microgravity-induced ophthalmic changes could affect astronauts’ visual capabilities and task performance, and some astronauts may experience persistent or permanent visual alterations even after returning to Earth^21^, presenting significant challenges for long-duration space exploration. Future human space exploration, specifically long-term missions, will require an increased inter-disciplinary understanding of the effects of the space environment on life.

In summary, more comprehensive research is needed for (1) revealing various biophysical phenomena in space, (2) understanding the effects of these phenomena on the physiology and health of life in spaceflight, and (3) the exploitation of these phenomena in the development of space-based biotechnologies. Using and continuously updating biophysical phenomena databases such as SpaceLID will provide the communities with unique detailed information for further analysis so that the ultimate goal—developing a method for mitigating the undesirable effects of space on life—can be reached.

## Data Availability

The article presents biophysical phenomena examples obtained using the SpaceLID system with all relevant data generated or research analysis results provided in the Study ID publications.

## References

[1] Witze, A. Astronauts have conducted nearly 3000 science experiments aboard the ISS. *Nature* (2020). 10.1038/d41586-020-03085-8 33149317

[2] Afshinnekoo E (2020). Fundamental biological features of spaceflight: Advancing the field to enable deep-space exploration. Cell.

[3] Wang J (2022). Database of space life investigations and bioinformatics of microbiology in extreme environments. Front. Microbiol..

[4] Ray S (2019). GeneLab: Omics database for spaceflight experiments. Bioinformatics.

[5] Beheshti A, McDonald JT, Miller J, Grabham P, Costes SV (2019). GeneLab database analyses suggest long-term impact of space radiation on the cardiovascular system by the activation of FYN through reactive oxygen species. Int. J. Mol. Sci..

[6] Polyakov VV, Lacota NG, Gundel A (2001). Human thermohomeostasis onboard "Mir" and in simulated microgravity studies. Acta Astronaut..

[7] Stahn AC (2017). Increased core body temperature in astronauts during long-duration space missions. Sci. Rep..

[8] Kumar S, Medale M, Marco PD, Brutin D (2020). Sessile volatile drop evaporation under microgravity. NPJ Microgravity.

[9] Fortney SM, Mikhaylov V, Lee SM, Kobzev Y, Gonzalez RR, Greenleaf JE (1998). Body temperature and thermoregulation during submaximal exercise after 115-day spaceflight. Aviat. Space Environ. Med..

[10] Morrison SF. Central control of body temperature. *F1000Res* **5**, (2016). 10.12688/f1000research.7958.1 PMC4870994 27239289

[11] Vessel, E. A. & Russo, S. Effects of Reduced Sensory Stimulation and Assessment of Countermeasures for Sensory Stimulation Augmentation. A Report for NASA Behavioral Health and Performance Research: Sensory Stimulation Augmentation Tools for Long Duration Spaceflight (NASA/TM-2015-218576). *NASA Center for AeroSpace Information*. (2015). https://osf.io/mvjxd?view_only=d051040205734a67826a1756aa0990d3.

[12] Otsuka K (2015). Intrinsic cardiovascular autonomic regulatory system of astronauts exposed long-term to microgravity in space: Observational study. NPJ Microgravity.

[13] Goldberger AL, Amaral LA, Hausdorff JM, Ivanov P, Peng CK, Stanley HE (2002). Fractal dynamics in physiology: Alterations with disease and aging. Proc. Natl. Acad. Sci. USA.

[14] Goncalves AJ, Braga MVA, Santana PH, Resende L, da Silva VJD, Correia D (2021). Linear and non-linear analysis of heart rate variability in HIV-positive patients on two different antiretroviral therapy regimens. BMC Infect. Dis..

[15] Aoyagi N, Ohashi K, Tomono S, Yamamoto Y (2000). Temporal contribution of body movement to very long-term heart rate variability in humans. Am. J. Physiol. Heart Circ. Physiol..

[16] Aoyagi N, Ohashi K, Yamamoto Y (2003). Frequency characteristics of long-term heart rate variability during constant-routine protocol. Am. J. Physiol. Regul. Integr. Comp. Physiol..

[17] Marshall-Goebel K (2019). Assessment of jugular venous blood flow stasis and thrombosis during spaceflight. JAMA Netw. Open.

[18] Roberts DR (2017). Effects of spaceflight on astronaut brain structure as indicated on MRI. N. Engl. J. Med..

[19] Lee JK (2019). Spaceflight-associated brain white matter microstructural changes and intracranial fluid redistribution. JAMA Neurol..

[20] Marshall-Bowman K, Barratt MR, Gibson CR (2013). Ophthalmic changes and increased intracranial pressure associated with long duration spaceflight: An emerging understanding. Acta Astronaut.

[21] Mader TH (2021). Persistent globe flattening in astronauts following long-duration spaceflight. Neuroophthalmology.

